# The effects of global postural re-education on sleep quality and stress in university women lecturers: a randomized controlled trial

**DOI:** 10.3389/fpsyt.2023.1321588

**Published:** 2024-01-16

**Authors:** Manuel Rodríguez-Aragón, David Barranco-Rodríguez, Marta de Mora-Martín, Sandra Sánchez-Jorge, David Varillas-Delgado, Noelia Valle-Benítez

**Affiliations:** ^1^Faculty of Health Sciences, Universidad Francisco de Vitoria, Madrid, Spain; ^2^Faculty of Experimental Sciences, Universidad Francisco de Vitoria, Madrid, Spain

**Keywords:** sleep quality, Pittsburg questionnaire, stress, cortisol, high education, self-treatment

## Abstract

**Objective:**

The present study aimed to evaluate the effect of global postural re-education (GPR) on sleep quality and stress in female health science lecturers.

**Methods:**

A total of 24 female university lecturers were allocated in this randomized controlled trial into intervention (*n* = 12) and control (*n* = 12) groups. The intervention group underwent familiarization and training on the therapy to execute an 8-week treatment with GPR. Data collected on sleep quality were analyzed using 24-h accelerometry (Actigraphy GT3X+) in addition to the Pittsburgh Sleep Quality Index (PSQI) questionnaire score as well as the State-Trait Anxiety Inventory (STAI) anxiety questionnaire. Data on stress were collected by measuring salivary cortisol.

**Results:**

After comparing the GPR of the groups, there was a main effect of the group (*F* = 5.278, *p* = 0.044) for PSQI. The *post-hoc* analysis revealed that both groups decreased scores between pre- and post-treatment. Additionally, post-treatment, there were differences between groups. For sleep latency, there were main effects of group (*F* = 6.118, *p* = 0.031) and score × group (*F* = 9.343, *p* = 0.011) interactions. The *post-hoc* analysis revealed that treatment groups decreased scores between pre- and post-treatment, and there were differences between groups (all *p* < 0.050).

**Conclusion:**

The self-administered GPR improves sleep quality in female university lecturers, providing a valuable self-regulation tool for enhanced sleep quality and enhanced academic performance. Further study may help to develop this as a potential tool to help university lecturers' job performance.

## 1 Introduction

Human beings need adequate control to regulate the stimuli received during his life and sufficient rest to be able to obtain a biological, physical, and emotional balance. In this sense, stress and sleep quality are influential determinants of health status (1). University lecturers are exposed to certain degrees of pressure related to personal management with students, other lecturers, administrative staff, and people in senior or management positions (2). The professional development of the university lecturer requires, in many cases, in addition to teaching, an added labor toward research, management, participation in talks and/or committees, extra training, work groups, supervision of students, maintenance with the profession of base, and pressure for coordination with different people from the university environment (3). The sum of these charges is a predisposing factor that damages the health of a person (4, 5). Similarly, due to the pandemic declared in 2020, consequences have been described for the mental and physical health of people, including stress and sleep quality (6). Adaptation to the pandemic situation has been associated with a decrease in the physical and mental wellbeing of lecturers (7).

Sleep is a physiological process defined as a behavior and state of the brain that appears daily. The circadian phases were adjusted to night and day (8) and were regulated in the suprachiasmatic nucleus of the hypothalamus (9). During this reversible state, awareness and response to the environment are diminished. Sleep problems affect a person's performance and health. Total or partial sleep deprivation can generate physiological and psychological changes such as attention deficits, irritability, motivation, and stress, among others (10).

Stress is an adaptive and necessary reaction that prepares the body to react to different situations. From the point of view of neuroscience, considered a type of emotional activation, stress is defined as a physiological reaction that affects the properties of brain cells and can affect the nervous system and other systems, as well as behavioral processes and cognitive processes (11). It has positive connotations since it is necessary to keep the individual alert, motivated, and strong. On the other hand, if stress occurs in excess and adaptation is not achieved, it paralyzes, generates anger, sadness, and fear, and can have negative repercussions on health (1, 12). In situations of stress, among other glucocorticoids, the adrenal glands secrete cortisol upon receiving adrenocorticotropin (ACTH) from the bloodstream. ACTH is produced after the activation of the hypothalamic-pituitary-adrenal axis and is driven into the pituitary by corticotropin produced in the hypothalamus (13).

Physiological processing in the face of mechanical and psychological stimuli is different in men and women. The prevalence of mental and physical pathologies has marked patterns according to gender. It is of special interest to study the differences between genders since gender is considered an important and influential biological factor in vulnerability to psychosocial stress (14). Women perceive potentially stressful circumstances as more intense than men, such as their job occupation or the social role they have acquired (15), showing a higher incidence of mood problems and anxiety states in women compared to men (16).

Global postural re-education (GPR) is a physiotherapy proposal that attempts to coordinate the tension of the muscular chains using the powerful components of concentration, flexibility, proprioception, and strength using guided breathing (17). It is a therapy of wide clinical use, and its effects have been studied in cervical pain (18), low back pain (19), and temporomandibular disorders (20). Similarly, it has been studied for other types of pathologies such as urinary incontinence (21, 22), ankylosing spondylitis (23), and even in Alzheimer's and Parkinson's diseases (24). Although GPR has been widely employed in clinical practice, demonstrating its utility in treating various pathologies (18–24), further research is needed to determine its effectiveness in other areas due to the variability of physiological implications triggered during its execution. GPR stands out as a fundamental pillar, particularly for its focus on slow and controlled respiratory mechanics. Laborde et al. (25) point out that the slow and voluntary breathing characteristic of GPR leads to an increase in parasympathetic nervous system control mediated by the vague nerve. This type of breathing, specifically abdominal or diaphragmatic, has been shown to improve sleep quality given its crucial role in the body. Similarly, abdominal breathing used in GPR is characterized by deep inhalations and prolonged exhalations, facilitating oxygen intake and carbon dioxide elimination and inducing bodily relaxation and a decrease in stress and anxiety levels (26). This effect promotes an improvement in sleep onset as well as in quality and restfulness during sleep (25).

In this context, there is a need for gaining knowledge of tools that reduce costs in national healthcare systems, enhance patient autonomy, and aim to support the reduction of inappropriate self-administration of medications focused on improving sleep and anxiety. Furthermore, these tools should be aimed at improving patient safety under the guidance and education of healthcare professionals, requiring minimal time for implementation, allowing flexibility, and adapting to the patient's lifestyle to facilitate adherence.

Therefore, the present study aimed to evaluate whether the application of a self-treatment program with GPR has effects on sleep quality and stress in higher education lecturers. We hypothesized that effective sleep quality in female university lecturers' conditions is good for academic performance and that self-treatment with GPR helps to improve sleep quality and stress in this cohort of female lecturers.

## 2 Materials and methods

### 2.1 Study design

A single-center, randomized, controlled study (National Clinical Trial identifier NTC05488015) was assessed.

### 2.2 Participants

A total of 24 female university lecturers from the Universidad Francisco de Vitoria, Madrid, Spain were included in this study. The inclusion criteria were (a) female university lecturers and (b) age between 32 and 61 years. The exclusion criteria were as follows: (a) pregnant university lecturers, who due to hormonal fluctuations, such as increased progesterone levels, can experience daytime sleepiness, frequent nighttime awakenings, and difficulties falling asleep, contributing to decreased sleep quality. Additionally, physical changes such as weight gain and physical discomfort can interfere with comfort during rest, thereby increasing stress levels; (b) those who use of drugs [non-steroidal anti-inflammatory drugs (NSAIDs), anticonvulsants, beta-blockers, and antidepressants]: These drugs can influence sleep quality and stress levels in various ways. For instance, antidepressants and beta-blockers may alter neurotransmitter regulation in the brain, affecting sleep patterns. In addition, anticonvulsants might induce daytime drowsiness or disrupt sleep cycles, negatively impacting rest quality; (c) those with musculoskeletal or neurological pathologies associated with sleep disorders such as chronic pain, discomfort, or involuntary movements during sleep, disrupting both sleep quality and quantity. Chronic pain can wake individuals up during the night and hinder their falling back asleep, contributing to the onset of sleep disorders; (d) those affected by sleep disorders such as sleep apnea disrupt breathing during sleep, leading to micro-awakenings and poor sleep quality. Circadian rhythm disorders affect the body's internal clock, making it challenging to regulate the sleep-wake cycle, causing insomnia or other sleep issues that may elevate stress levels; (e) those with acute or subacute back pain or pathology can cause significant discomfort at night, frequent awakenings, and difficulty finding a comfortable sleeping position, thereby disrupting sleep and increasing stress levels; and (f) those with tumors and rheumatological, adrenal, or pituitary diseases. These conditions can directly impact sleep quality and stress levels due to physical symptoms such as pain, fatigue, or hormonal changes that affect the ability to rest adequately and manage stress effectively.

All participants signed a written informed consent before participating in the study. The study protocol was approved by the Research Ethics Committee of the Universidad Francisco de Vitoria (UFV 18/2021), following the Declaration of Helsinki of 1964 (last actualization: 2013).

### 2.3 Sample size

The sample size was calculated using G^*^Power 3.4 software (27). An *a priori* sample size calculation indicated that female students of health sciences were needed to obtain statistically significant differences between the intervention and control groups. This *a priori* sample size was calculated to obtain an effect size of 25.7% of reduction in stress by using the GPR method (statistical power of 80% with type I error set at 5%) based on a previous investigation that obtained these results when they studied the effect of the intervention regarding the control group in the intervention group (28). A target sample size of 24 participants was determined.

### 2.4 Randomization method

Participants were randomly allocated after baseline data collection in a parallel group (1:1 ratio) to either an intervention group or a control group. Participants were randomized on an Excel-generated randomization schedule. The research coordinators regularly performed data quality control, management, and protocol compliance verification. Due to the nature of the intervention, blinding of participants, care providers, and outcome assessors was not possible.

### 2.5 Procedure

The experimental group was provided with different materials to become familiar with the intervention. These materials were as follows: (1) an informative triptych with the GPR intervention process and with the positions to be carried out explained in writing and images; (2) two explanatory videos on the execution and evolution of the postures; (3) an audio that allows to follow the evolution of the postures step by step during their execution, with the necessary corrections to avoid making mistakes; (4) an exam that allowed us to evaluate and establish the knowledge about the learning obtained; and (5) the participants had the continuous support of the researchers during the intervention time.

#### 2.5.1 Intervention

The GPR postures were done before going to sleep for 4–5 days a week for 8 weeks. The two chosen postures were performed on the ground, looking for a hard and stable surface and unloading gravity to facilitate self-management. The first position was a coxofemoral opening with closed arms. The position begins with the participant lying supine on the floor, with the arms open at 90° and the palms of the hands facing the ceiling. The lower limbs begin with hip and knee flexion, with the soles of the feet together, the heels near the gluteal region, and a hip abduction opening between 30° and 45°. The physiological curves of the spine must be maintained, and good support and alignment between the occiput and the sacrum are sought. From that starting position, the posture progressively evolves, seeking to close the arms along the body and extend the hips and knees in the direction of an anatomical position. The second position to perform was coxofemoral closure with closed arms. This position begins with the participant lying supine, with arms open at 90° and palms facing the ceiling. The lower limbs are found with the hips and knees flexed and the soles of the feet together and leaning against a wall. The physiological curves must be maintained, and good support and alignment of the occipital and sacral regions are sought. The posture evolves by progressively closing the arms toward the patient's body and raising the legs up the wall, generating tension in the posterior myofascial chain. During the execution of both postures, the participants had to maintain a specific GPR breath as the fundamental basis of the method. Each posture was held for 15 min, with a total of 30 min per session.

### 2.6 Outcomes and measurements

The participants received personal instructions to collect the different samples and fill out the different questionnaires and outcomes as follows:

#### 2.6.1 Primary outcomes

##### 2.6.1.1 Actigraphy

Actigraphy GT3X+ [*ActiGraph, (Pensacola, FL, EEUU*)] activity bracelets were used to take measurements of the quality of sleep. Accelerometry data were collected using a 90-Hz sampling rate and integrated into 60-s steps (24, 29). The participants wore the bracelet during the week before the intervention to collect baseline measurements and the week after the intervention for comparison. The bracelets were maintained for a full week at each shot (30). It was indicated that the bracelet should be worn on the non-dominant hand (31). The data provided by the bracelets were as follows: In Bed (time to go to bed), Out Bed (time to get up), Latency (time spent from going to bed to falling asleep in minutes), Efficiency (the efficiency of sleep itself, according to the hours spent sleeping and being in bed), total time in bed (TTB), total sleep time (TST—total sleep time expressed in minutes), wake after sleep onset (WASO—time of awakenings while asleep), Number of awakenings at night, and Average Awakening (average time of each awakening at night). The results obtained were extracted from a computer using the Actylife version 6.9 software.

##### 2.6.1.2 Pittsburgh self-administered Sleep Quality Index questionnaire

Actigraphy data were supported by the self-administered Pittsburgh self-administered Sleep Quality Index (PSQI) Questionnaire (32). The PSQI is a validated questionnaire, and it has been shown to report reliable measures (33). The PSQI consists of 19 questions that assess sleep factors such as sleep duration, frequency, or latency. These questions are grouped into seven proficiency scores and are rated from 0 (no difficulty) to 3 (great difficulty). These seven scores report on other components of subjective sleep quality, such as subjective quality, sleep latency, sleep duration, habitual sleep efficiency, sleep disturbances, use of hypnotic medication, and daytime dysfunction. For the final score, seven scores are added, with the highest scores being those with the worst sleep quality (32).

##### 2.6.1.3 Sleep diary

Self-reported sleep quality with a sleep diary was used. This diary was filled out every day of the study, including the weeks in which the baseline measurements were taken before and after the intervention. Participants were informed and reminded in the mornings that they had to fill in the diary about the night before (34, 35). The diary includes questions related to the quality of sleep, answered using a Likert scale, a question to verify the performance of the posture during the week, and a section for observations. The diary allows the calculation of certain parameters: TST, sleep onset latency at the beginning of the night (SOL), WASO, sleep efficiency (SE), and perceived sleep quality or satisfaction using the Likert scale established from 0 (very poor) to 4 (very good) (35, 36).

#### 2.6.2 Secondary outcomes

##### 2.6.2.1. Salivary cortisol level

To collect the amount of cortisol in saliva as an indicator of stress levels, the participants were instructed about explanatory videos on the collection process. The Salivette^®^ kit (Sardtedt AG & Co. KG, Nümbrecht, Germany) was used. The saliva samples were analyzed by chemiluminescence after centrifuging for 2 min, following the manufacturer's instructions for the kit (Cortisol Saliva ELISA SA E-6000) from LDN^®^, which is developed and approved for the measurement of cortisol levels in saliva in humans.

The participants collected saliva for a full day to assess the circadian changes in cortisol levels, obtaining four samples: (1) upon waking up, (2) at 11:00 a.m., (3) at 3:00 p.m., and (4) at bedtime (37). The samples were stored at 4°C for no more than 2 days by the participants, and once they were delivered to the researchers, they were stored at −80°C until analysis.

##### 2.6.2.2 State-Trait Anxiety Inventory questionnaire

Anxiety is one of the emotional reactions to stress. The State-Trait Anxiety Inventory (STAI) questionnaire has been validated and is accepted by the scientific community to measure anxiety and relate it to stress levels. The questionnaire evaluates two independent concepts of anxiety: (a) state anxiety (STAI S-A), which is an individual's emotional and transitory anxiety condition, and (b) trait anxiety (STAI T-A), which is the individual's stable propensity for anxiety, which may be a trait of his or her personality (38). Each of these concepts include 20 items in the questionnaire. Each of the items is evaluated on a 4-point scale (0, not at all; 1, somewhat; 2, quite a bit; and 3, a lot), which is rated as inverse (if they decrease anxiety) or direct (if they increase anxiety) (38).

#### 2.6.3 Statistical analysis

Statistical analyses were performed using IBM Statistical Package for the Social Sciences (SPSS) Statistics for Windows, version 25.0 (IBM Corp., Armonk, United States). Continuous data were presented as the mean and standard deviation (SD) and 95% confidence intervals (95% CIs) of the mean. Continuous data were given as counts and percentages. The Shapiro-Wilk test was used to check the normality of all variables. Since all variables were normally distributed, parametric tests were applied to examine differences among conditions. Differences in continuous data between groups were assessed with the *t*-test. The time courses of continuous variables were evaluated using a two-way analysis of variance (ANOVA) with a repeated measurements design, providing readings of a continuous quantity (dependent variable) at two levels of a within-subject factor and a dichotomous characteristic (e.g., group assignment) as an independent, between-subjects factor. Interactions between the results of a biomarker decline between pre-intervention and post-intervention examinations in the treated group and the control group were analyzed (e.g., results of a biomarker decline between pre- and post-intervention examinations in the treated group, whereas they stagnated or even rose in the control group). Finally, repeated measurement of variance analysis was conducted to identify potential interaction effects between time and sessions in the study outcomes as follows. To determine whether participants' anxiety significantly changed and to uncover potential differences between groups at pre- and post-assessment, the STAI questionnaire was subjected to statistical analysis. Responses to the State and Trait Anxiety Inventory were scored separately to reveal an STAI S-A and STAI T-A, PSQI, TST, WASO, SE, and SOL. Each one was subjected to two-factor mixed repeated measures ANOVA (2 groups × 2 evaluation times). When a significant F value was obtained for any main effect or interaction, a least significant difference (LSD) *post-hoc* analysis was performed to determine pairwise differences for the values obtained pre- and post-treatment within each group. The significance level was set at a *p*-value of < 0.05.

## 3 Results

**Figure 1:**
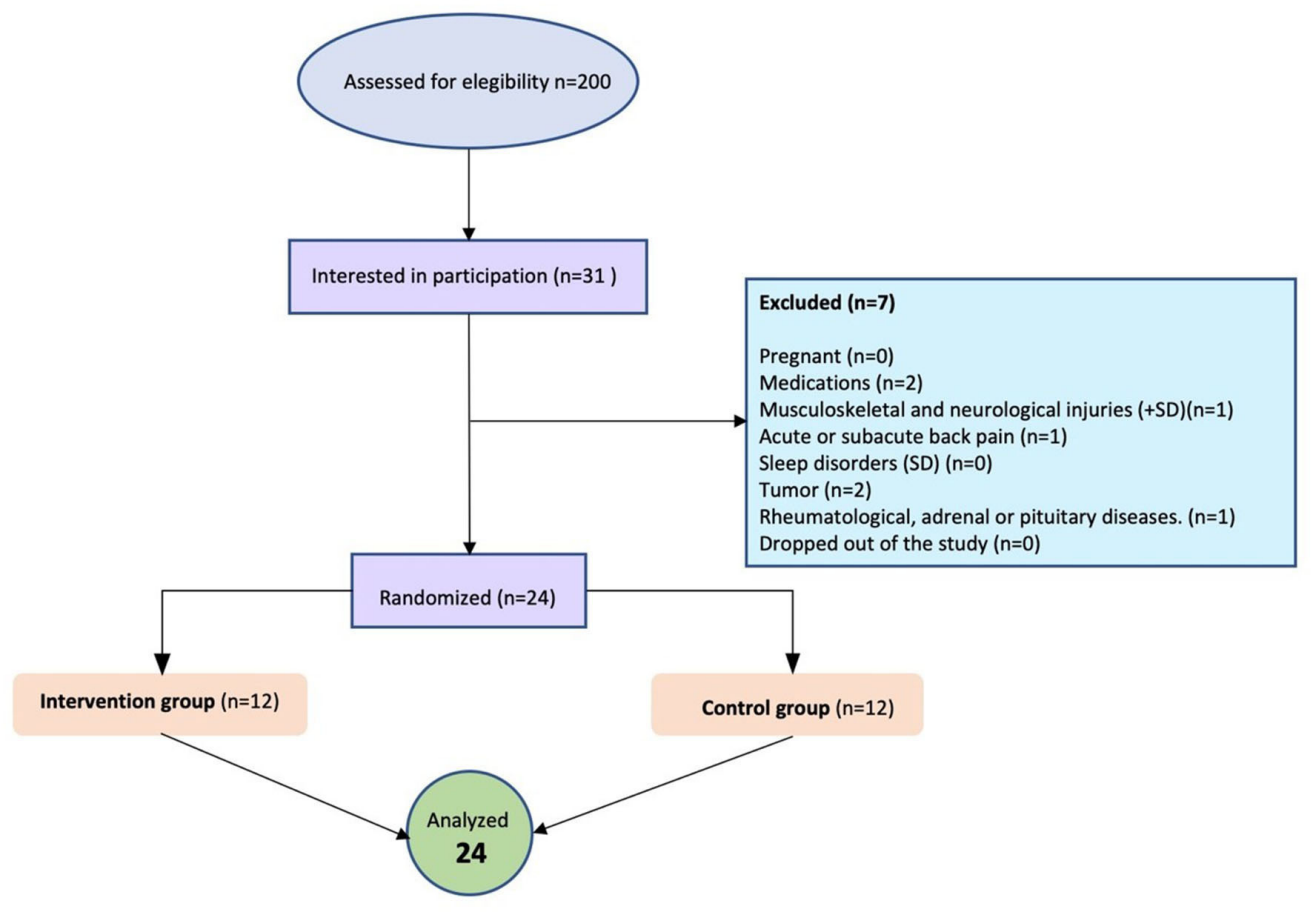
Flow diagram.

From September to October 2022, 31 women filled out the registration questionnaire to participate. A total of 24 participants meeting the inclusion criteria were contacted and recruited and randomized in the intervention and control groups. The sample was finally composed of 24 women lectures, 12 in the control group and 12 in the intervention group, who completed pre- and post-intervention assessments. Microsoft Excel software was used to randomize the participants and divide them into two groups, namely, the control group (*n* = 12) and the experimental group (*n* = 12; Figure 1).

Detailed baseline data for participants are shown in Table 1. As intended, both groups did not significantly differ regarding age, body mass index (BMI), biomarkers, and outcome scores.

**Table 1:** Baseline characteristics of both the intervention and control groups were presented by mean (standard deviation).

	**Intervention group**	**Control group**	**Effect size**	***p*-value**
Age (years)	38.08 (3.80)	42.42 (6.62)	0.542	0.105
Weight (kg)	58.83 (9.04)	63.37 (14.02)	0.385	0.365
Height (m)	1.65 (0.06)	1.65 (0.06)	0.016	0.973
BMI (kg/m^2^)	21.46 (2.74)	23.18 (5.48)	0.398	0.339
**Biomarker**				
Saliva cortisol (ng/dl)	19.81 (7.82)	20.53 (7.26)	0.094	0.827
**Outcomes**				
Sleep latency	4.09 (3.28)	2.12 (2.32)	−0.693	0.085
TST (min)	393.92 (45.04)	383.93 (43.36)	−0.225	0.587
WASO (min)	41.48 (31.59)	46.48 (25.62)	0.173	0.675
Awakenings	13.99 (4.58)	14.768 (6.611)	0.137	0.74
Average awakenings	2.90 (1.77)	3.20 (1.37)	0.187	0.649
TIB (min)	440.21 (51.47)	431.94 (35.72)	−0.186	0.652
STAI (T-A)	33.08 (25.03)	32.75 (29.08)	−0.012	0.976
STAI (S-A)	40.83 (25.88)	39.17 (28.39)	−0.061	0.882
PSQI	5.00 (3.07)	5.58 (2.91)	0.194	0.638

BMI, body mass index; STAI (T-A), State-Trait Anxiety Inventory Trait Anxiety; STAI (S-A), State-Trait Anxiety Inventory State Anxiety; PSQI, Pittsburgh; WASO, wakefulness after the onset of sleep; SE, sleep efficiency; TST, total sleep time; SOL, sleep onset latency at the beginning of night sleep.

**Figure 2:**
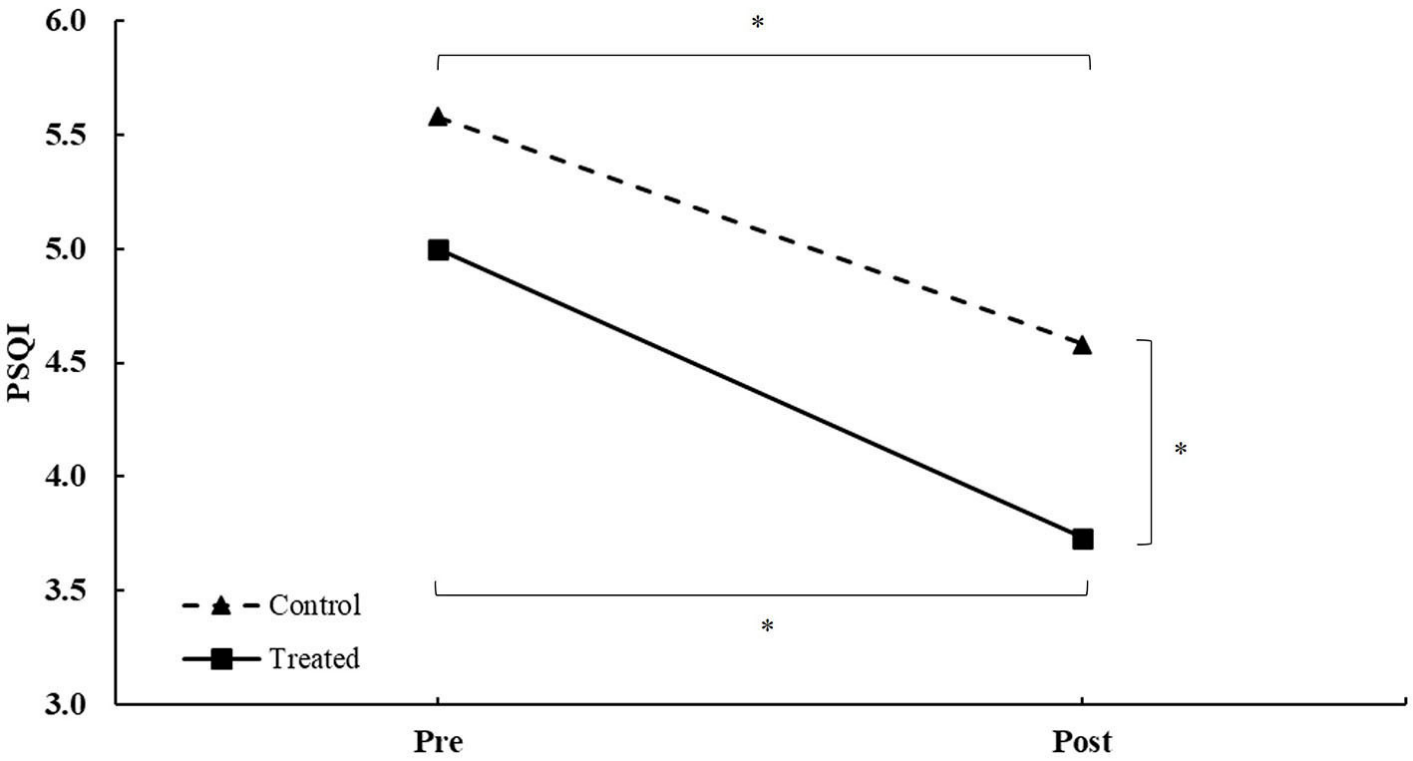
PSQI score during intervention in patients between the treated and control groups during intervention. *Differences at a *p*-value of < 0.050.

For PSQI, there was a main effect of group (*F* = 5.28, *p* = 0.044) with no main effects of the score (*F* = 0.67, *p* = 0.433) and score × group (*F* = 0.00, *p* = 0.952) interaction (Table 2). The *post-hoc* analysis revealed that both groups decreased scores between pre- and post-treatment. Additionally, post-treatment, there were differences between groups (all *p* < 0.050; Figure 2).

**Table 2:** Data on repeated measurement design and differences in outcome scores between the intervention and control groups.

	**Main effect score**	**Main effect group**	**Interaction group × score**
Sleep latency	*F* = 0.53	*F* = 6.12	*F* = 9.34
	df_1_ = 1; df_2_ = 11	df_1_ = 1; df_2_ = 11	df_1_ = 1; df_2_ = 11
	Partial η^2^ = 0.046	Partial η^2^ = 0.357	Partial η^2^ = 0.459
	*p* = 0.482	*p* = 0.031^*^	*p* = 0.011^*^
TST (min)	*F* = 2.59	*F* = 1.33	*F* = 0.89
	df_1_ = 1; df_2_ = 11	df_1_ = 1; df_2_ = 11	df_1_ = 1; df_2_ = 11
	Partial η^2^ = 0.206	Partial η^2^ = 0.117	Partial η^2^ = 0.082
	*p* = 0.138	*p* = 0.276	*p* = 0.367
WASO (min)	*F* = 0.42	*F* = 0.00	*F* = 0.00
	df_1_ = 1; df_2_ = 11	df_1_ = 1 df_2_ = 11	df_1_ = 1; df_2_ = 11
	Partial η^2^ = 0.037	Partial η^2^ = 0.000	Partial η^2^ = 0.000
	*p* = 0.531	*p* = 0.944	*p* = 0.969
Awakenings	*F* = 0.03	*F* = 0.85	*F* = 0.30
	df_1_ = 1; df_2_ = 11	df_1_ = 1 df_2_ = 11	df_1_ = 1; df_2_ = 11
	Partial η^2^ = 0.003	Partial η^2^ = 0.072	Partial η^2^ = 0.005
	*p* = 0.866	*p* = 0.376	*p* = 0.823
Average awakenings	*F* = 0.27	*F* = 0.29	*F* = 0.03
	df_1_ = 1; df_2_ = 11	df_1_ = 1 df_2_ = 11	df_1_ = 1; df_2_ = 11
	Partial η^2^ = 0.024	Partial η^2^ = 0.026	Partial η^2^ = 0.003
	*p* = 0.614	*p* = 0.602	*p* = 0.871
TIB (min)	*F* = 2.31	*F* = 0.900	*F* = 1.24
	df_1_ = 1; df_2_ = 11	df_1_ = 1 df_2_ = 11	df_1_ = 1; df_2_ = 11
	Partial η^2^ = 0.177	Partial η^2^ = 0.075	Partial η^2^ = 0.101
	*p* = 0.153	*p* = 0.364	*p* = 0.289
STAI (T-A)	*F* = 0.42	*F* = 2.62	*F* = 1.33
	df_1_ = 1; df_2_ = 11	df_1_ = 1 df_2_ = 11	df_1_ = 1; df_2_ = 11
	Partial η^2^ = 0.037	Partial η^2^ = 0.192	Partial η^2^ = 0.108
	*p* = 0.523	*p* = 0.134	*p* = 0.273
STAI (S-A)	*F* = 0.37	*F* = 2.99	*F* = 4.46
	df_1_ = 1; df_2_ = 11	df_1_ = 1 df_2_ = 11	df_1_ = 1; df_2_ = 11
	Partial η^2^ = 0.416	Partial η^2^ = 0.214	Partial η^2^ = 0.298
	*p* = 0.554	*p* = 0.112	*p* = 0.058
PSQI	*F* = 0.67	*F* = 5.28	*F* = 0.00
	df_1_ = 1; df_2_ = 11	df_1_ = 1 df_2_ = 11	df_1_ = 1; df_2_ = 11
	Partial η^2^ = 0.063	Partial η^2^ = 0.345	Partial η^2^ = 0.000
	*p* = 0.433	*p* = 0.044^*^	*p* = 0.952

df, degrees of freedom; TST, total sleep time; WASO, wake after sleep onset; STAI (T-A), Trait Anxiety Inventory; STAI (S-A), State Anxiety Inventory; PSQI, Pittsburgh. Effect sizes are given as partial η^2^. Statistically significant ^*^p < 0.05.

**Figure 3:**
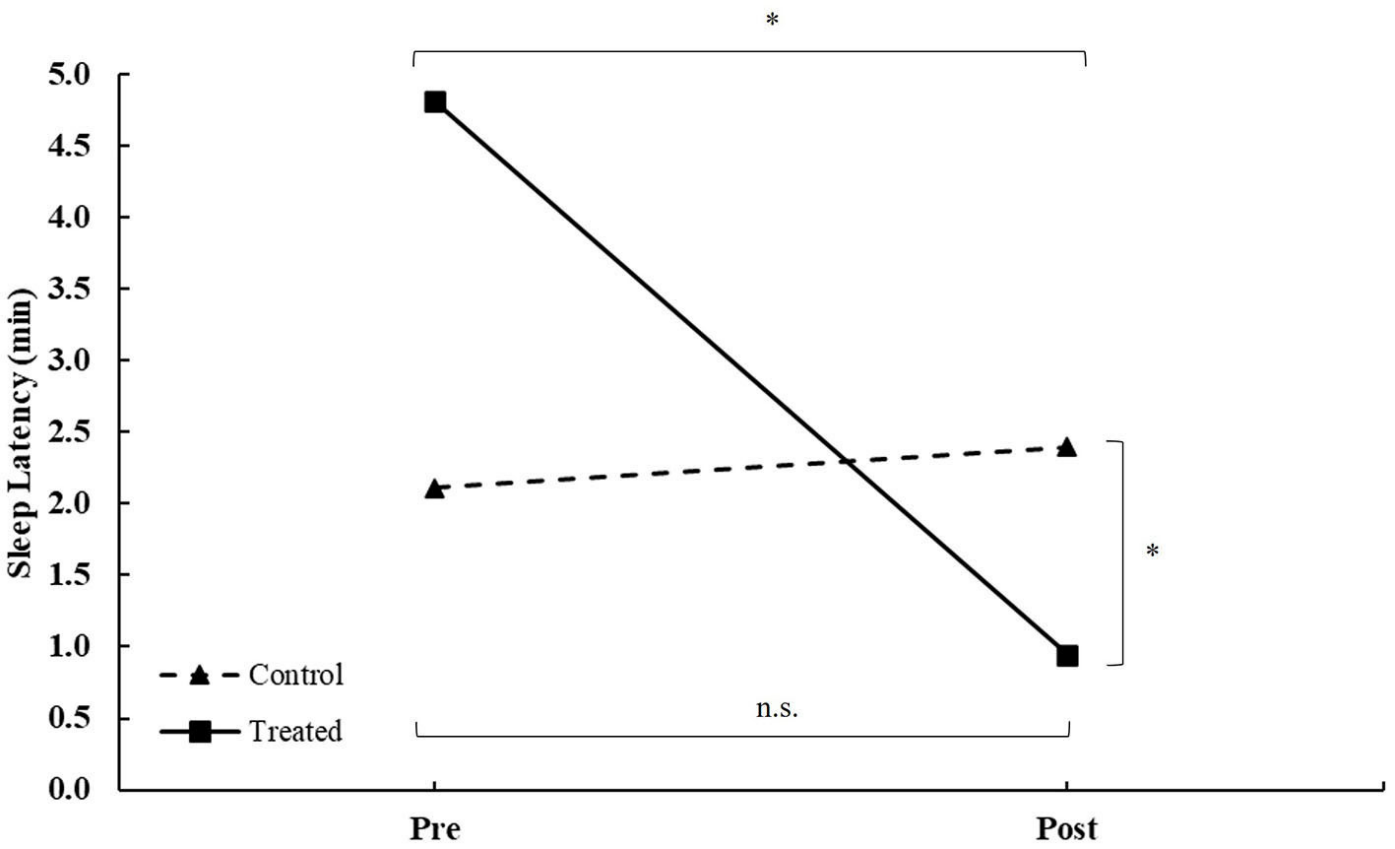
Sleep latency during intervention in patients between the treated and control groups during intervention. *Differences at a *p*-value of < 0.050, n.s., non-significant.

For sleep latency, there was a main effect of group (*F* = 6.12, *p* = 0.031) and score × group (*F* = 9.34, *p* = 0.011) interaction, with no main effect of the score (*F* = 0.53, *p* = 0.482; Table 2). The *post-hoc* analysis revealed that the treatment group decreased scores between pre- and post-treatment. Additionally, post-treatment, there were differences between groups (all *p* < 0.050; Figure 3).

## 4 Discussion

This study evaluates the application of a self-treatment program with GPR effects on sleep quality and stress among university lecturers, showing the first outcomes during 8 weeks of intervention on sleep quality and stress in female university lecturers. The results obtained from the GPR through the PSQI questionnaire and actigraphy suggest that the intervention had a positive effect on the sleep quality of this cohort of women lecturers.

Previous studies have shown that light to moderate physical activity, done in the morning or in the evening, has had a positive impact on sleep quality even in interventions of shorter duration than the present study (39, 40). Along the same lines, there are studies that apply other physical activities, such as yoga, which requires concentration, posture maintenance, and guided breathing. These studies have also demonstrated their effectiveness in improving sleep quality in older adults (41–43).

It is also possible that the positive effects of GPR on sleep quality are due to the reduction of muscular tensions and the improvement of body posture that this therapy has already demonstrated from a musculoskeletal point of view through the performance of specific exercises that seek muscular balance and the release of muscular tensions (18, 19). Several studies have linked the improvement of posture and the reduction of muscular tensions with a relaxing effect on the body and, therefore, may favor a better quality of sleep (44, 45).

It is interesting to note that both the sleep diary and actigraphy measure some similar variables, allowing the results between the two tests to be compared from subjective and objective points of view. Overall, this study found a good correlation between the data collected by the two tests, although there were also some notable differences. For example, actigraphy revealed that some participants slept less than their sleep diary indicated, which could indicate an overestimation in the diary. This could be due to a greater awareness of their sleep and a more critical subjective assessment of sleep quality, whereas actigraphy only measures objective aspects such as sleep duration and amount of movement. These discrepancies between objective and subjective sleep assessment have already been exposed in other studies (46, 47). It was also suggested that actigraphy might not be sensitive enough to detect subtle improvements in sleep quality (48).

On the other hand, the positive effects on sleep quality in this study may have been positively influenced by the time of the intervention. Studies such as those by Tsai et al. (49) not only perform the intervention before sleep but also focus on guided breathing that helps to decrease vagal activity. In other words, one could attribute to GPR a relaxing and meditative nature that reduces the physiological and mental activity necessary to improve sleep quality. In any case, it is important to keep in mind that the effects of GPR on sleep quality may depend on several factors, such as the duration and frequency of the intervention, as well as the individual characteristics of the participants, such as their age, gender, and stress level. In this sense, from a methodological point of view, this study is aligned with the recommendations of intervention with GPR used in different studies such as Amorim et al. (50), Brooks et al. (51), and Kloek et al. (52). However, we can contrast the results with other studies focused on older people, children, or special populations and using interventions of different durations ranging from 6 weeks (53) to 12 weeks (54). Therefore, it would be interesting to expand GPR studies focusing on physiological and cognitive variables such as sleep quality and stress to determine the best protocols. It is important to note that the sample used in this study is composed exclusively of female university lecturers, which limits the generalizability of the results to other populations. However, these findings provide relevant information for understanding sleep quality in this specific group and may be useful for the implementation of intervention programs aimed at improving sleep quality in women university lecturers.

The relationship between sleep quality and stress has been previously evaluated in female university lecturers in studies such as that of Vela-Bueno et al. (55), where they found that poor sleep quality associated with the professionals evaluated was associated with a greater presence of job stress and lower job satisfaction, which is highly prevalent in university lecturers (56). In view of this relationship, stress was also assessed in this study using the biomarker cortisol and the STAI anxiety questionnaire. For these variables, the proposed intervention does not seem to have had different effects if we compare the results of the control group with those of the intervention group. Although there are studies such as the one by Sugano and Nomura (57) that claim that stretching can have a positive effect on cortisol control, there are different factors that may influence why this study did not obtain the same results. It is possible that the 8-week intervention was not sufficient to generate significant changes in cortisol levels in female university lecturers. Some studies have found that stress reduction through therapies such as meditation and yoga can take longer than 8 weeks to be evident in salivary cortisol levels (58, 59). On the other hand, the heterogeneity of risk factors that play a role in stress among university lecturers may have limited the effectiveness of the stress intervention (60). Given the close relationship between students and lecturers and their mutual influence throughout the academic year, conducting a study involving students to compare stress levels, sleep patterns, and the effects of such interventions between both populations would be highly valuable. Exploring these aspects in students could provide a comprehensive understanding of the broader educational environment and potentially yield insights into strategies for enhancing overall wellbeing and academic performance across the entire educational community.

The overarching goals of the World Health Organization's (WHO) health-promoting universities program emphasize a comprehensive approach to health encompassing physical, mental, and social wellbeing within academic settings (61). Universities are increasingly aware of the significance of faculty wellbeing and the strategies needed to achieve it. This study aligns with these strategies, focusing on university professors but potentially transferable to the wider university community. GPR not only targets physical aspects such as posture or pain reduction but also addresses fundamental psychological aspects such as stress management and sleep improvement. It signifies an enhancement of both physical and mental health, contributing to cultivating a healthier academic environment. Furthermore, the methodology employed provides self-care tools, promoting health and a balance between work and personal life among university lecturers.

This investigation exhibits certain strengths that merit emphasis. It can be asserted that the measurement and comparison of variables using objective markers and subjective assessments through questionnaires have allowed us to highlight the therapeutic intention focused on patient perception and preferences. Moreover, interventions that can be appropriately guided may empower patients with significant autonomy and contribute to cost savings for National Healthcare Systems. However, this study presents potential limitations. We can enumerate the following: (a) the small sample size in this study raises caution when making definitive conclusions due to the small sample size; (b) the aspect of self-management by patients, which could potentially benefit from therapist-assisted manual treatment amplification; and (c) the absence of in-person familiarization sessions. Incorporating in-person sessions with therapist-guided management during the familiarization process would be beneficial or, following classroom-based training/treatment, live lessons in between self-management would be beneficial. Given these limitations, the findings of this investigation should be extrapolated to subjects following a similar familiarization and treatment methodology.

## 5 Conclusion

This randomized controlled study shows for the first time that GPR self-treatment helps to improve sleep quality in female university lecturers and will serve as a support in periods of need so that this autonomy can serve as a catalyst that will result in improved sleep quality and, consequently, better psychological conditions to enhance work performance.

Based on these findings, it seems worthwhile to encourage and promote research among other populations, particularly those related to educational settings, who may also experience sleep quality problems and stress, such as university students (62). Exploration of these aspects by students could provide an overall understanding of the broader educational environment and potentially yield insights into strategies for improving general wellbeing and academic performance across the educational community.

Similarly, it will be interesting to apply these results in future studies to people suffering from diseases that are highly correlated with sleep quality problems, such as patients with arterial hypertension (63).

## Data availability statement

The raw data supporting the conclusions of this article will be made available by the authors, without undue reservation.

## Ethics statement

The study protocol was approved by the Research Ethics Committee of the Universidad Francisco de Vitoria (UFV 18/2021), following the Declaration of Helsinki of 1964 (last actualization: 2013). All participants signed a written informed consent before participating in the study.

## Author contributions

MR-A: Conceptualization, Funding acquisition, Investigation, Methodology, Visualization, Writing – review & editing. DB-R: Data curation, Methodology, Writing – review & editing. MM-M: Data curation, Methodology, Writing – review & editing. SS-J: Data curation, Methodology, Writing – review & editing. DV-D: Formal analysis, Investigation, Methodology, Software, Visualization, Writing – original draft, Writing – review & editing. NV-B: Conceptualization, Investigation, Methodology, Visualization, Writing – review & editing.

## References

[1] Navinés R Martín-Santos R Olivé V Valdés M. Work-related stress: implications for physical and mental health. Med Clin. (2016) 146:359–66. 10.1016/j.medcle.2016.06.010 26806767

[2] Cardozo LT Azevedo MAR Carvalho MSM Costa R de Lima PO Marcondes FK. Effect of an active learning methodology combined with formative assessments on performance, test anxiety, and stress of university students. Adv Physiol Educ. (2020) 44:744–51. 10.1152/advan.00075.2020 33205996

[3] Huda BZ Rusli BN Naing L Tengku MA Winn T Rampal KG. A study of job strain and dissatisfaction among lecturers in the School of Medical Sciences Universiti Sains Malaysia. Southeast Asian J Trop Med Public Health. (2004) 35:210–8. 15272771

[4] Nakata A. Psychosocial job stress and immunity: a systematic review. Methods Mol Biol. (2012) 934:39–75. 10.1007/978-1-62703-071-7_3 22933140

[5] Siegrist J. Adverse health effects of high-effort/low-reward conditions. J Occup Health Psychol. (1996) 1:27–41. 10.1037/1076-8998.1.1.27 9547031

[6] Almeida ILL Rego JF Teixeira ACG Moreira MR. Social isolation and its impact on child and adolescent development: a systematic review. Rev Paul Pediatr. (2021) 40:e2020385. 10.1590/1984-0462/2022/40/2020385 34614137 PMC8543788

[7] Idris F Zulkipli IN Abdul-Mumin KH Ahmad SR Mitha S Rahman HA. Academic experiences, physical and mental health impact of COVID-19 pandemic on students and lecturers in health care education. BMC Med Educ. (2021) 21:542. 10.1186/s12909-021-02968-2 34702230 PMC8548144

[8] Moore RY. Neural control of the pineal gland. Behav Brain Res. (1996) 73:125–30. 10.1016/0166-4328(96)00083-6 8788489

[9] Rosenwasser AM Turek FW. Neurobiology of circadian rhythm regulation. Sleep Med Clin. (2015) 10:403–12. 10.1016/j.jsmc.2015.08.003 26568118

[10] Killgore WD. Effects of sleep deprivation on cognition. Prog Brain Res. (2010) 185:105–29. 10.1016/B978-0-444-53702-7.00007-5 21075236

[11] Pruessner M Pruessner JC Hellhammer DH Bruce Pike G Lupien SJ. The associations among hippocampal volume, cortisol reactivity, and memory performance in healthy young men. Psychiatry Res. (2007) 155:1–10. 10.1016/j.pscychresns.2006.12.007 17395434

[12] Folkman S. The case for positive emotions in the stress process. Anxiety Stress Coping. (2008) 21:3–14. 10.1080/10615800701740457 18027121

[13] McEwen BS. Physiology and neurobiology of stress and adaptation: central role of the brain. Physiol Rev. (2007) 87:873–904. 10.1152/physrev.00041.2006 17615391

[14] Wang J Korczykowski M Rao H Fan Y Pluta J Gur RC. Gender difference in neural response to psychological stress. Soc Cogn Affect Neurosci. (2007) 2:227–39. 10.1093/scan/nsm018 17873968 PMC1974871

[15] Asher M Asnaani A Aderka IM. Gender differences in social anxiety disorder: a review. Clin Psychol Rev. (2017) 56:1–12. 10.1016/j.cpr.2017.05.004 28578248

[16] Steel Z Marnane C Iranpour C Chey T Jackson JW Patel V. The global prevalence of common mental disorders: a systematic review and meta-analysis 1980-2013. Int J Epidemiol. (2014) 43:476–93. 10.1093/ije/dyu038 24648481 PMC3997379

[17] Dischiavi SL Wright AA Hegedus EJ Bleakley CM. Biotensegrity and myofascial chains: a global approach to an integrated kinetic chain. Med Hypotheses. (2018) 110:90–6. 10.1016/j.mehy.2017.11.008 29317079

[18] Cunha AC Burke TN França FJ Marques AP. Effect of global posture reeducation and of static stretching on pain, range of motion, and quality of life in women with chronic neck pain: a randomized clinical trial. Clinics. (2008) 63:763–70. 10.1590/S1807-59322008000600010 19060998 PMC2664276

[19] Merinero D Rodríguez-Aragón M Álvarez-González J López-Samanes Á López-Pascual J. Acute effects of global postural re-education on non-specific low back pain. does time-of-day play a role? Int J Environ Res Public Health. (2021) 15:18. 10.3390/ijerph18020713 33467603 PMC7829940

[20] Monteiro W Francisco de Oliveira Dantas da Gama T dos Santos RM Collange Grecco LA Pasini Neto H Oliveira CS. Effectiveness of global postural reeducation in the treatment of temporomandibular disorder: case report. J Bodyw Mov Ther. (2013) 17:53–8. 10.1016/j.jbmt.2012.05.003 23294684

[21] Fozzatti C Herrmann V Palma T Riccetto CL Palma PC. Global postural re-education: an alternative approach for stress urinary incontinence? Eur J Obstet Gynecol Reprod Biol. (2010) 152:218–24. 10.1016/j.ejogrb.2010.06.002 20638774

[22] Fozzatti MC Palma P Herrmann V Dambros M. [Impact of global postural reeducation for treatment of female stress urinary incontinence]. Rev Assoc Med Bras. (2008) 54:17–22. 10.1590/S0104-42302008000100015 18392481

[23] Coksevim NH Durmus D Kuru O. Effects of global postural reeducation exercise and anti-TNF treatments on disease activity, function, fatigue, mobility, sleep quality and depression in patients with active ankylosing spondylitis: a prospective follow-up study. J Back Musculoskelet Rehabil. (2018) 31:1005–12. 10.3233/BMR-170901 30412478

[24] Sadeh A Sharkey KM Carskadon MA. Activity-based sleep-wake identification: an empirical test of methodological issues. Sleep. (1994) 17:201–7. 10.1093/sleep/17.3.201 7939118

[25] Laborde S Allen MS Borges U Dosseville F Hosang TJ Iskra M. Effects of voluntary slow breathing on heart rate and heart rate variability: a systematic review and a meta-analysis. Neurosci Biobehav Rev. (2022) 138:104711. 10.1016/j.neubiorev.2022.104711 35623448

[26] Lee JS Lee MS Lee JY Cornélissen G Otsuka K Halberg F. Effects of diaphragmatic breathing on ambulatory blood pressure and heart rate. Biomed Pharmacother. (2003) 57(Suppl 1):87s−91s. 10.1016/j.biopha.2003.08.011 14572682

[27] Faul F Erdfelder E Lang AG Buchner A. G^*^Power 3: a flexible statistical power analysis program for the social, behavioral, and biomedical sciences. Behav Res Methods. (2007) 39:175–91. 10.3758/BF03193146 17695343

[28] Gao L Zhang D Wang S Jia Y Wang H Sun X. Effect of the app-based video guidance on prenatal pelvic floor muscle training combined with global postural re-education for stress urinary incontinence prevention: a protocol for a multicenter, randomized controlled trial. Int J Environ Res Public Health. (2021) 8:18. 10.3390/ijerph182412929 34948546 PMC8700899

[29] Cole RJ Kripke DF Gruen W Mullaney DJ Gillin JC. Automatic sleep/wake identification from wrist activity. Sleep. (1992) 15:461–9. 10.1093/sleep/15.5.461 1455130

[30] Eiman MN Pomeroy JML Weinstein AA. Relationship of actigraphy-assessed sleep efficiency and sleep duration to reactivity to stress. Sleep Sci. (2019) 12:257–64. 10.5935/1984-0063.20190090 32318246 PMC7159077

[31] Ozemek C Kirschner MM Wilkerson BS Byun W Kaminsky LA. Intermonitor reliability of the GT3X+ accelerometer at hip, wrist and ankle sites during activities of daily living. Physiol Meas. (2014) 35:129–38. 10.1088/0967-3334/35/2/129 24399138

[32] Buysse DJ Reynolds CF 3rd Monk TH Berman SR Kupfer DJ. The Pittsburgh Sleep Quality Index: a new instrument for psychiatric practice and research. Psychiatry Res. (1989) 28:193–213. 10.1016/0165-1781(89)90047-4 2748771

[33] Jiménez-Genchi A Monteverde-Maldonado E Nenclares-Portocarrero A Esquivel-Adame G de la Vega-Pacheco A. [Reliability and factorial analysis of the Spanish version of the Pittsburg Sleep Quality Index among psychiatric patients]. Gac Med Mex. (2008) 144:491–6. 19112721

[34] Carney CE Buysse DJ Ancoli-Israel S Edinger JD Krystal AD Lichstein KL. The consensus sleep diary: standardizing prospective sleep self-monitoring. Sleep. (2012) 35:287–302. 10.5665/sleep.1642 22294820 PMC3250369

[35] Ibáñez V Silva J Cauli O. A survey on sleep questionnaires and diaries. Sleep Med. (2018) 42:90–6. 10.1016/j.sleep.2017.08.026 29458752

[36] Salfi F Lauriola M Tempesta D Calanna P Socci V De Gennaro L. Effects of total and partial sleep deprivation on reflection impulsivity and risk-taking in deliberative decision-making. Nat Sci Sleep. (2020) 12:309–24. 10.2147/NSS.S250586 32547280 PMC7261660

[37] Dmitrieva NO Almeida DM Dmitrieva J Loken E Pieper CF. A day-centered approach to modeling cortisol: diurnal cortisol profiles and their associations among U.S. adults. Psychoneuroendocrinology. (2013) 38:2354–65. 10.1016/j.psyneuen.2013.05.003 23770247 PMC3776005

[38] Guillén-Riquelme A Buela-Casal G. [Meta-analysis of group comparison and meta-analysis of reliability generalization of the State-Trait Anxiety Inventory Questionnaire (STAI)]. Rev Esp Salud Publica. (2014) 88101:−12. 10.4321/S1135-57272014000100007 24728394

[39] Benloucif S Orbeta L Ortiz R Janssen I Finkel SI Bleiberg J. Morning or evening activity improves neuropsychological performance and subjective sleep quality in older adults. Sleep. (2004) 27:1542–51. 10.1093/sleep/27.8.1542 15683146

[40] Naylor E Penev PD Orbeta L Janssen I Ortiz R Colecchia EF. Daily social and physical activity increases slow-wave sleep and daytime neuropsychological performance in the elderly. Sleep. (2000) 23:87–95. 10.1093/sleep/23.1.1f 10678469

[41] Halpern J Cohen M Kennedy G Reece J Cahan C Baharav A. Yoga for improving sleep quality and quality of life for older adults. Altern Ther Health Med. (2014) 20:37–46. 24755569

[42] Hariprasad VR Sivakumar PT Koparde V Varambally S Thirthalli J Varghese M. Effects of yoga intervention on sleep and quality-of-life in elderly: a randomized controlled trial. Indian J Psychiatry. (2013) 55:S364–368. 10.4103/0019-5545.116310 24049200 PMC3768213

[43] Shree Ganesh HR Subramanya P Rao MR Udupa V. Role of yoga therapy in improving digestive health and quality of sleep in an elderly population: a randomized controlled trial. J Bodyw Mov Ther. (2021) 27:692–7. 10.1016/j.jbmt.2021.04.012 34391308

[44] Akodu AK Akindutire OM. The effect of stabilization exercise on pain-related disability, sleep disturbance, and psychological status of patients with non-specific chronic low back pain. Korean J Pain. (2018) 31:199–205. 10.3344/kjp.2018.31.3.199 30013734 PMC6037811

[45] Finan PH Goodin BR Smith MT. The association of sleep and pain: an update and a path forward. J Pain. (2013) 14:1539–52. 10.1016/j.jpain.2013.08.007 24290442 PMC4046588

[46] Backhaus J Junghanns K Broocks A Riemann D Hohagen F. Test-retest reliability and validity of the Pittsburgh Sleep Quality Index in primary insomnia. J Psychosom Res. (2002) 53:737–40. 10.1016/S0022-3999(02)00330-6 12217446

[47] Sohn SI Kim DH Lee MY Cho YW. The reliability and validity of the Korean version of the Pittsburgh Sleep Quality Index. Sleep Breath. (2012) 16:803–12. 10.1007/s11325-011-0579-9 21901299

[48] Nishikawa K Kuriyama K Yoshiike T Yoshimura A Okawa M Kadotani H. Effects of cognitive behavioral therapy for insomnia on subjective-objective sleep discrepancy in patients with primary insomnia: a small-scale cohort pilot study. Int J Behav Med. (2021) 28:715–26. 10.1007/s12529-021-09969-x 33629218

[49] Tsai HJ Kuo TB Lee GS Yang CC. Efficacy of paced breathing for insomnia: enhances vagal activity and improves sleep quality. Psychophysiology. (2015) 52:388–96. 10.1111/psyp.12333 25234581

[50] Amorim CS Gracitelli ME Marques AP Alves VL. Effectiveness of global postural reeducation compared to segmental exercises on function, pain, and quality of life of patients with scapular dyskinesis associated with neck pain: a preliminary clinical trial. J Manipulative Physiol Ther. (2014) 37:441–7. 10.1016/j.jmpt.2013.08.011 25092553

[51] Brooks SK Webster RK Smith LE Woodland L Wessely S Greenberg N. The psychological impact of quarantine and how to reduce it: rapid review of the evidence. Lancet. (2020) 395:912–20. 10.1016/S0140-6736(20)30460-8 32112714 PMC7158942

[52] Kloek CJJ van Dongen JM de Bakker DH Bossen D Dekker J Veenhof C. Cost-effectiveness of a blended physiotherapy intervention compared to usual physiotherapy in patients with hip and/or knee osteoarthritis: a cluster randomized controlled trial. BMC Public Health. (2018) 18:1082. 10.1186/s12889-018-5975-7 30170586 PMC6119267

[53] Sheikhi B. Effect of global postural reeducation exercise on pain and hip muscle flexibility in patients with chronic low back pain and movement control dysfunction. Int J Basic Sci Med. (2019) 4:148–54. 10.34172/ijbsm.2019.05

[54] Lawand P Lombardi Júnior I Jones A Sardim C Ribeiro LH Natour J. Effect of a muscle stretching program using the global postural reeducation method for patients with chronic low back pain: a randomized controlled trial. Joint Bone Spine. (2015) 82:272–7. 10.1016/j.jbspin.2015.01.015 25881758

[55] Vela-Bueno A Moreno-Jiménez B Rodríguez-Muñoz A Olavarrieta-Bernardino S Fernández-Mendoza J De la Cruz-Troca JJ. Insomnia and sleep quality among primary care physicians with low and high burnout levels. J Psychosom Res. (2008) 64:435–42. 10.1016/j.jpsychores.2007.10.014 18374744

[56] Huda BZ Rusli BN Naing L Winn T Tengku MA Rampal KG. Job strain and its associated factors among lecturers in the School of Medical Sciences, Universiti Sains Malaysia and Faculty of Medicine, Universiti Kebangsaan Malaysia. Asia Pac J Public Health. (2004) 16:32–40. 10.1177/101053950401600106 18839865

[57] Sugano A Nomura T. Influence of water exercise and land stretching on salivary cortisol concentrations and anxiety in chronic low back pain patients. J Physiol Anthropol Appl Human Sci. (2000) 19:175–80. 10.2114/jpa.19.175 11037691

[58] Curtis K Osadchuk A Katz J. An eight-week yoga intervention is associated with improvements in pain, psychological functioning and mindfulness, and changes in cortisol levels in women with fibromyalgia. J Pain Res. (2011) 4:189–201. 10.2147/JPR.S22761 21887116 PMC3160832

[59] Pascoe MC Thompson DR Ski CF. Yoga, mindfulness-based stress reduction and stress-related physiological measures: a meta-analysis. Psychoneuroendocrinology. (2017) 86:152–68. 10.1016/j.psyneuen.2017.08.008 28963884

[60] Wang P Chu P Wang J Pan R Sun Y Yan M. Association between job stress and organizational commitment in three types of chinese university teachers: mediating effects of job burnout and job satisfaction. Front Psychol. (2020) 11:576768. 10.3389/fpsyg.2020.576768 33132985 PMC7578428

[61] Tsouros A Dowding G Thomson J Dooris M. Health Promoting Universities: Concept, Experience and Framework for Action. Copenhagen: World Health Organization Regional Office for Europe (1998)

[62] Suardiaz-Muro M Morante-Ruiz M Ortega-Moreno M Ruiz MA Martín-Plasencia P Vela-Bueno A. [Sleep and academic performance in university students: a systematic review]. Rev Neurol. (2020) 71:43–53. 10.33588/rn.7102.2020015 32627159

[63] Han B Chen WZ Li YC Chen J Zeng ZQ. Sleep and hypertension. Sleep Breath. (2020) 24:351–6. 10.1007/s11325-019-01907-2 31402441 PMC7127991

